# Anoxic metabolism and biochemical production in *Pseudomonas putida* F1 driven by a bioelectrochemical system

**DOI:** 10.1186/s13068-016-0452-y

**Published:** 2016-02-18

**Authors:** Bin Lai, Shiqin Yu, Paul V. Bernhardt, Korneel Rabaey, Bernardino Virdis, Jens O. Krömer

**Affiliations:** Centre for Microbial Electrochemical Systems (CEMES), The University of Queensland, Office 618, Gehrmann Building (60), St. Lucia, Brisbane, QLD 4072 Australia; Advanced Water Management Centre (AWMC), The University of Queensland, Brisbane, Australia; School of Chemistry and Molecular Biosciences, The University of Queensland, Brisbane, Australia; Laboratory of Microbial Ecology and Technology (LabMET), Ghent University, Ghent, Belgium

**Keywords:** Anoxic metabolism, *Pseudomonas putida* F1, Bioelectrochemical system, Redox mediators, Extracellular electron transfer, Bio-production, Chemical feedstocks

## Abstract

**Background:**

*Pseudomonas putida* is a promising host for the bioproduction of chemicals, but its industrial applications are significantly limited by its obligate aerobic character. The aim of this paper is to empower the anoxic metabolism of wild-type *Pseudomonas putida* to enable bioproduction anaerobically, with the redox power from a bioelectrochemical system (BES).

**Results:**

The obligate aerobe *Pseudomonas putida* F1 was able to survive and produce almost exclusively 2–Keto-gluconate from glucose under anoxic conditions due to redox balancing with electron mediators in a BES. 2-Keto-gluconate, a precursor for industrial anti-oxidant production, was produced at an overall carbon yield of over 90 % based on glucose. Seven different mediator compounds were tested, and only those with redox potential above 0.207 V (vs standard hydrogen electrode) showed interaction with the cells. The productivity increased with the increasing redox potential of the mediator, indicating this was a key factor affecting the anoxic production process. *P. putida* cells survived under anaerobic conditions, and limited biofilm formation could be observed on the anode’s surface. Analysis of the intracellular pools of ATP, ADP and AMP showed that cells had an increased adenylate energy charge suggesting that cells were able to generate energy using the anode as terminal electron acceptor. The analysis of NAD(H) and NADP(H) showed that in the presence of specific extracellular electron acceptors, the NADP(H) pool was more oxidised, while the NAD(H) pool was unchanged. This implies a growth limitation under anaerobic conditions due to a shortage of NADPH and provides a way to limit biomass formation, while allowing cell maintenance and catalysis at high purity and yield.

**Conclusions:**

For the first time, this study proved the principle that a BES-driven bioconversion of glucose can be achieved for a wild-type obligate aerobe. This non-growth bioconversion was in high yields, high purity and also could deliver the necessary metabolic energy for cell maintenance. By combining this approach with metabolic engineering strategies, this could prove to be a powerful new way to produce bio-chemicals and fuels from renewables in both high yield and high purity.

**Electronic supplementary material:**

The online version of this article (doi:10.1186/s13068-016-0452-y) contains supplementary material, which is available to authorized users.

## Background

Contrary to renewable fuels, which have steadily increased their share in the energy sector [1], bulk and speciality chemicals are still mainly derived from petroleum and natural gas. However, industrial biotechnology has significantly developed over the recent decades, and it now offers more solutions for the sustainable production of chemicals from renewable resources than ever before [2]. A range of products are currently produced in biotechnological processes [3], including enzymes, amino acids, antibiotics, alcohols, organic acids and vitamins using ever-expanding range of evolved and genetically engineered microorganisms. Such biotechnological processes, however, often face limitations based on redox balance, carbon yields or product toxicity.

A class of microbes that was recently recognised as a promising new platform for the production of chemical feedstocks (often toxic even to the microbial production strain) are the pseudomonads [4]. They have been used to produce antimicrobial aromatics such as phenol [5] and show, in comparison with other industrial organisms such as *Escherichia coli* or baker’s yeast, particular advantages in solvent tolerance [6, 7]. This allows higher product concentrations of compounds with solvent properties [8–11]. In this family, *Pseudomonas putida* (*P. putida*) is regarded a model strain for studying the catabolism and synthesis of toxic aromatics. A range of aromatic compounds, such as 3-methylcatechol [12] and p-hydroxybenzoic acid [13], have been produced with *P. putida.*

*Pseudomonas putida* is a gram-negative, rod-shaped, flagellated, saprotrophic soil bacterium that is frequently isolated from soil contaminated with petrochemicals. It relies on oxygen as terminal electron acceptor and does not ferment [4, 14]. *Pseudomonas* catabolism is efficient in the supply of redox power [15], but with a low cellular energy demand needed for cell maintenance; in other words, it has a high net NAD(P)H generation for enzymatic reactions [16]. In *P. putida* under aerobic conditions on glucose, the most important sources for NADPH are the pentose-phosphate pathway (PPP), and Entner-Doudoroff (ED) pathway enzymes glucose-6-phosphate dehydrogenase and phosphogluconate dehydrogenase [17, 18]. Because NADPH is an important co-factor for metabolite biosynthesis [19] and dealing with toxicity [15], *P. putida* has been described as one of the promising new platform organisms adaptable for biotechnology and synthetic biology applications [4].

The strictly aerobic metabolism of *P. putida*, however, may also lead to complications when it comes to industrial applications. On the one hand, it increases the capital cost, as scaling-up of aerobic process is significantly limited by the oxygen transfer rate [20], and due to this, both the maximum and average scales of commercial aerobic fermenters are much smaller in comparison with anaerobic fermenters [21]. On the other hand, aerobic production is also inseparable from substrate loss in the form of CO_2_, while some anaerobic processes can achieve carbon yields close to 100 % [22]. To overcome these limitations, a range of studies of *P. putida* under oxygen-limiting conditions have been conducted aiming at developing an anaerobic mutant of *P. putida* [23]. However, the success to date has been limited, and so far only a reduced death rate of *P. putida* cells in anaerobic conditions could be achieved while limited catabolic activity was observed [18, 24].

To solve the problem of electron balancing and allow efficient anaerobic metabolism of *P. putida*, we proposed to culture the organism in the anodic compartment of a bioelectrochemical system (BES). BES were firstly proposed as technology for electricity production from biodegradable waste [25–27] and then extended to other applications including hydrogen production [28], water desalination [29], nutrient removal and recovery [30–33] as well as bio-production [30] including the conversion of CO_2_ to acetate [34] and methane [35]. BESs use electrodes as electron sink or donor to drive the anoxic metabolism of microbial cells. In a recently reported study, electroactivity under different oxygen-limited conditions was observed for a recombinant *P. putida* KT2440 [36]. This bacterium was engineered to produce pyocyanin, an electron shuttle (or mediator molecule) from *Pseudomonas aeruginosa* [37].

Under the rationale that an electrode can act as electron acceptor (sink) in order to balance the intracellular energy and redox co-factors, in the present study, we investigate the mediated electron transport in a non-genetically modified *P. putida* F1 under strictly anaerobic conditions with glucose as a carbon source. We observed electrochemical activity and high yield production of 2-Keto-gluconate (2KGA), which is an industrial precursor for the production of antioxidant iso-ascorbic acid [38]. We used various mediators with broad electrochemical midpoint potentials and tested for electrochemical activity in the presence of the microbial cells. Using quantitative metabolite analysis, we show the impact of the presence of an extracellular electron acceptor on intracellular energy balance as well as the co-factor ratios of NAD^+^/NADH and NADP^+^/NADPH.

## Methods

### Strain and cultivation conditions

The strain used in this study was wild-type *P. putida* F1. Cells were cultivated in a defined mineral medium (DM9) that contained per litre: 6 g Na_2_HPO_4_, 3 g KH_2_PO_4_, 0.1 g NH_4_Cl, 0.1 g MgSO_4_·7H_2_O, 15 mg CaCl_2_·2H_2_O and 1 ml trace element solution, containing per litre: 1.5 g FeCl_3_·6H_2_O, 0.15 g H_3_BO_3_, 30 mg CuSO_4_·5H_2_O, 0.18 g KI, 0.12 g MnCl_2_·4H_2_O, 60 mg Na_2_MoO_4_·2H_2_O, 0.12 g ZnSO_4_·7H_2_O, 0.15 g CoCl_2_·6H_2_O, 10 g EDTA (acid), and 23 mg NiCl_2_·6H_2_O. Glucose was used as the sole metabolic carbon source in all tests, and the initial medium pH was 7. Cultivation temperature was 30 °C [39]. Cell density was analysed photometrically using absorbance at 600 nm (OD_600_) and converted to cell dry weight (CDW) by the following empirically determined conversion factor: CDW [g/L] = 0.476 × OD_600_.

Pre-cultures were prepared by picking and transferring of a single colony from a LB plate into baffled shake flasks for aerobic overnight cultivation (~16 h) in an orbital shaking incubator (2.5 cm orbit, Multitron, Infors, Bottmingen, Switzerland) at 200 rpm and 30 °C. When cell density reached OD_600_ = 0.5 (in log phase) cells were harvested by centrifugation (7000*g*, room temperature, 10 min), washed, resuspended in fresh DM9 medium, and then transferred into the main cultivations vessels. Anaerobic tests were done in 150 mL anaerobic culture bottles or BES reactors. Anaerobic conditions throughout the experiments were assured by sparging the culture medium with nitrogen. The anaerobic bottles were inoculated in an anaerobic chamber.

### Bioelectrochemical system set up

The double-chamber BESs consisted of a double-jacketed sterilisable glass vessel with a net liquid volume of 350 mL serving as the anodic compartment. The cathodic compartment consisted of a 15 mL glass cannula inserted directly into the anode chamber. A circular cation exchange membrane (diameter 9 mm, CMI-7000, Membranes International INC., USA) was mounted at the bottom of the cannula to guarantee ionic connection between anodic and cathodic electrolytes. Additional file 1: Fig. S1 in the supporting information depicts a schematic of the BESs. Carbon cloth (projected surface area of ~25 cm^2^) was used as the anode electrode after pre-treatment with a modified cetyltrimethylammoniumbromide (CTAB) soaking method [40]. In brief, the carbon cloth electrodes were soaked in 2 mM CTAB solution and incubated in a shaking incubator at 40 °C, 200 rpm for 16 h. The pre-treatment was necessary to clean the carbon cloth and to improve the hydrophilicity. A titanium mesh (Kaian Metal Wire Mesh, Anping, P.R. China) was used as cathode electrode. An Ag/AgCl electrode in saturated KCl (+0.197 V vs standard hydrogen electrode) was used as reference electrode (Cat. 013457, RC-1CP, Als, Tokyo, Japan), and titanium wire (T555518, Advent Research Materials, Oxford, United Kingdom) was used as electric wire for all connections. For ease of comparison, all potentials herein are reported with respect to the standard hydrogen electrode (SHE). The working electrode potential was controlled to a set potential relatively to the reference electrode using a potentiostat (Potentiostat/Galvanostat VSP, BioLogic Science Instruments, France).The potentials were chosen based on the electrochemical characterisation of the mediators (vide infra). Riboflavin, [Co(Sep)]Cl_3_, Fe(EDTA), thionine chloride, Co(bpy)_3_](ClO_4_)_2_, K_3_[Fe(CN)_6_] were added (1 mM) as redox mediators to the culture medium. These mediators cover a wide range of electrochemical midpoint potentials and were tested for electrochemical activity in the presence of the microbial cells. Measurements of current production over time (chronoamperometry) were used to monitor the electrocatalytic activity of the microbes. Strictly anaerobic conditions were maintained by flushing sterile nitrogen gas through the reactor headspace at a flow rate of around 30 mL/min. Dissolved oxygen concentration was confirmed to be below detection limit (<15 ppb) using an optical oxygen sensor (OXY-4 mini, PreSens, Regensburg, Germany) in preliminary tests. A condenser cooled with 4 °C H_2_O was used to reduce water evaporation through the headspace due to the flushing.

### Analytics and sampling

The concentrations of glucose, gluconic acid, 2KGA and acetic acid were analysed using an Agilent 1200 high performance liquid chromatography (HPLC) system and an Agilent Hiplex H column (300 × 7.7 mm, PL1170–6830) with guard column (SecurityGuard Carbo-H, Phenomenex PN: AJO-4490). In brief, the column temperature was set to 40 °C, and analytes were eluted isocratically with 14 mM H_2_SO_4_ at a flow rate of 0.4 mL/min. Glucose was detected using a refractive index detector, while the carboxylates were detected with absorbance at 210 nm. Prior to injection, culture broth was collected by centrifugation at 16,000 g, 4 °C for 10 min, and the supernatant was used for HPLC analysis undiluted.

### Cell extraction for intracellular metabolite analysis

Samples were taken from the electrochemical reactors between 73 and 100 h when the current reached its peak value. In the controls, samples were taken at the similar time point as above when mediators were fully reduced.

For the analysis of intracellular ATP/ADP/AMP, cell pellets representing between 0.2 and 0.4 mg CDW were harvested by centrifugation at 12,000*g*, 4 °C for 2 min. Cell extractions for NAD(P) +/NAD(P)H were performed with a modified fast filtration—freeze/drying process [41, 42]. In brief, around 5 mg CDW were harvested by fast filtration (GVWP04700, 0.22-µm pore size, Millipore, Australia) and then quickly soaked into cold methanol solution (60 % v/v, −48 °C) and incubated at −48 °C for 20 min. The precise amount of CDW harvested was calculated from the optical density (as explained above) of the sample volume.

Afterwards, the extract was centrifuged at 10,000*g*, −4°C for 10 min (5810R, Eppendorf, Hamburg, Germany). Supernatants were collected and frozen in −80 °C freezer after dilution with high-purity water (R > 18 MΩ) to a final concentration of methanol <=20 % v/v). The frozen samples were lyophilised by a freeze-dryer at −20 °C and then resuspended into the reaction buffers provided by the enzymatic assay kits respectively.

For ATP/ADP/AMP analysis cell pellets were resuspended in 250 µL of ice-cold phosphate buffer (pH 7.75). Then cells were extracted by the cold trichloroaceticacid (TCA) method [17, 43]: addition of 250 μL ice-cold 5 % (w/v) TCA—4 mM EDTA and mixing (vortex, 20 s)and then incubation on ice for 20 min. Cell debris was removed by centrifugation (12,000*g*, 4 °C, 10 min) and the supernatant transferred to a new tube and kept on ice until subsequent analysis. Quantification of ATP content was conducted by a commercial bio-luminescence assay kit (LBR-S010, Biaffin, Germany) according to the manufacturer’s instructions. The bioluminescence signal was quantified using a microplate reader (M200, Tecan, Switzerland) with white 96-well plate (655075, Greiner Bio-one, Germany). Prior to the assay, samples were diluted between 5 and 40 fold in 20 mM Tris-H_2_SO_4_ buffer (pH 7.75) containing 2 mM EDTA, for two reasons: (1) to achieve readings within the calibration curve of the assay and (2) to dilute TCA present in the extracts. TCA interferes with the optical signals and by diluting it, the assay becomes less inhibited, and the inhibitory effect can be numerically corrected (Additional file 1: Fig. S2). Quantification of ADP and AMP were determined indirectly by enzymatically converting them to ATP as described previously [44]. ADP was converted to ATP using pyruvate kinase (P9136, Sigma) while adenylate kinase (M5520, Sigma) was added in addition to also convert AMP. The reaction mixture was incubated at 37 °C for 15 min. Concentrations of ADP and AMP were calculated based on the difference in luminescent reading of samples with or without enzymatic conversions. Adenylate energy charge (AEC) was then calculated according to the formula:

$${\text{AEC }} = {{\left( {{\text{ATP}} + 0. 5\times {\text{ADP}}} \right)} \mathord{\left/ {\vphantom {{\left( {{\text{ATP}} + 0. 5\times {\text{ADP}}} \right)} {{\text{ATP}} + {\text{ADP}} + {\text{AMP}}}}} \right. \kern-0pt}( {{\text{ATP}} + {\text{ADP}} + {\text{AMP}}})}$$

NADP^+^/NADPH were quantified using a commercial colorimetric assay kit (MAR038, Sigma-Aldrich, USA), and NAD^+^/NADH was determined by a fluorimetric assay kit (PicoProbe™, K338-100, BioVision, USA) according to manufacturer’s instructions.

### Electrochemical analysis and calculations

The midpoint redox potentials of the soluble redox mediators were determined by cyclic voltammetry (CV) in a two–chambered electrochemical cell filled with medium containing 0.1 M KCl (counter electrode chamber) and pH 7.0 0.1 M phosphate buffer (working electrode chamber). During the measurements, the potential of the working electrode (a 2 cm^2^ CTAB pre-treated carbon cloth) was swept between a low and a high limit (within a potential window between 0.9 and 1.0 V vs SHE) at a scan rate of 50 m V s^−1^. Measurements were repeated for at least 100 cycles to guarantee reproducibility. A graphite rod was used as the counter electrode, while a reference electrode was placed into the anode chamber. During the tests, individual mediators were added at a concentration of 1 mM. The midpoint potential values (*E*_m_) were determined as the arithmetic averages of the anodic (*E*_pa_) and cathodic peak (*E*_pc_) potentials as determined during the forward and backward scans of the CVs, respectively, according to equation $E_{\text{m}} =(E_{\rm{pa}}+E_{\rm{pc}})/2$.

In order to provide sufficient driving force for the oxidation of the redox mediator during normal reactor operations (chronoamperometry), the working electrode was set to a potential more positive (about 0.3 V) than the mid-point potentials as determined by CV. Control experiments with mediator but without cells did not lead to oxidation of glucose (Additional file 1: Fig. S7).

The coulombic efficiency (CE), i.e. the efficiency in the transfer of electric charge during the conversions was determined using the equation:

$${\text{CE [\%] }} = {Y_{\text{electrons}}/(Y_{\text{2KGA}}\times 4+{Y_{\text{acetic acid}}}\times 4 +Y_{\text{gluconic acid}}\times 2)}\times100$$

where *Y* is the molar yield coefficient on glucose basis of the products (electrons, 2KGA, acetic acid, gluconic acid, etc.) multiplied by the number of electrons released during the formation of the respective product from glucose. Per mol of glucose 4 mol electrons are generated when 2KGA is formed, 2 mol electrons are generated when gluconic acid is formed, and 4 mol electrons are generated when glucose is converted to CO_2_ and acetic acid. The molar yield coefficients were determined as the slope of a plot of mol product versus mol substrate converted (Additional file 1: Fig. S3). Reactor volume was corrected for withdrawn sample and water evaporation determined to be 0.09 mL/h at the reported headspace flushing rate.

The carbon balance was determined using the following equation:

$${\text{CB }}[ \%] \, = \, \left( {r_{{ 2 {\text{KGA}}}} \times 6+ \, {r_{\text{acetic acid}}} \times 2+ \, {r_{\text{gluconic acid}}} \times 6+ \, {r_{\text{CO}}}}_2 \right)/( {r_{\text{glucose}} \times 6}) \times 100$$

where *r* represents the specific uptake (glucose) or production rates (2KGA, acetic acid, gluconic acid and CO_2_) in [mmol/(g_CDW_ h)] and multiplied by the number of carbon atoms in the respective compound.

The production of CO_2_ could not be quantified because of CO_2_ evolution being miniscule compared to the rate of N_2_ flushing of the reactor headspace. However, the formation of acetate from pyruvate by *P. putida* will coincide with CO_2_ formation, either due to the activity of the membrane-bound decarboxylating pyruvate dehydrogenase/oxidase (EC 1.2.5.1) or to the activities of other metabolic reactions such as the formation of acetyl-CoA, which can be converted to acetate either through catabolic pathways or through biosynthesis processes. The ratio of CO_2_ to acetate in all these circumstances will be 1:1. Therefore the CO_2_ yield was assumed to be equal to the acetate yield, major shortcomings of the carbon balance would then point to high activity of pathways producing CO_2_, such as the TCA cycle or PPP. All product yield coefficients were converted to specific rates using the average planktonic biomass concentration in the systems. In fact, the contribution to the yields from cells forming biofilm was considered negligible.

## Results and discussion

### Redox mediators with high midpoint potential enable anoxic metabolism of *P. putida* F1

**Fig. 1:**
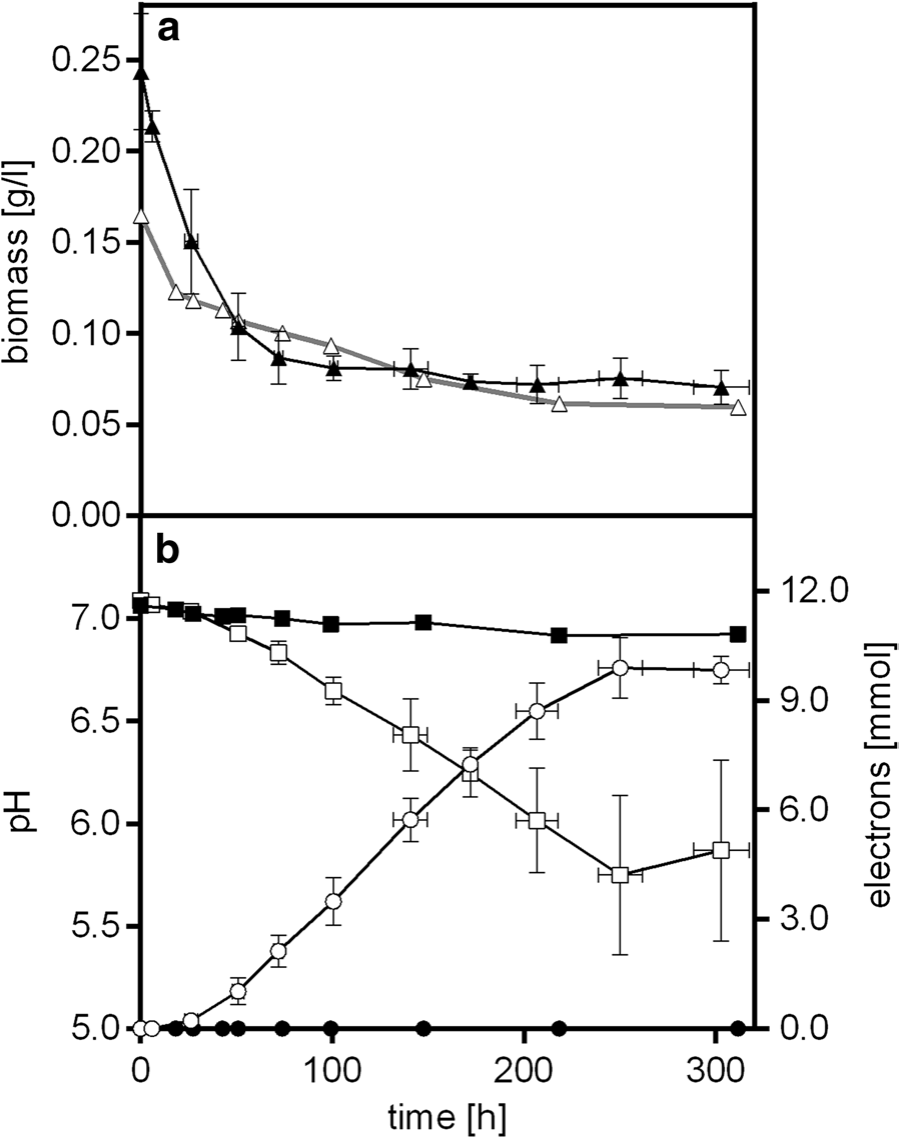
Change of biomass (*triangles*, **a**), pH (*squares*, **b**) and electron production (*circles*, **b**) in the anode compartment of a BES reactor of *P. putida* F1 with K_3_[Fe(CN)_6_] as electron acceptor in control (*black symbols*) and closed circuit with the anode potential poised at +0.697 V (*white symbols*). Data have been averaged from ten (closed circuit) and three (control) biological replicates with a total of 79 and 30 samples, respectively. Means and standard deviations (X and Y *error bars*) are given [average sample size *n* = 7 (closed circuit); exact sample size *n* = 3 (control)]

Like many of its family members, *P. putida* F1 is an obligate aerobe, and no anoxic growth in glucose-based mineral medium without oxygen is observed (Fig. 1a). It was previously shown that ferricyanide could serve as electron acceptor during the oxidation of nicotinic acid by *Pseudomonas fluorescens* [45]. In fact, when the glucose-based mineral medium (DM9) was supplemented with 1 mM potassium ferricyanide as electron acceptor, *P. putida* F1 anaerobically reduced ferricyanide (oxidised form [Fe(CN)_6_]^3−^) to ferrocyanide (reduced form, [Fe(CN)_6_]^4−^) within 55–90 h, as suggested by a change in colour of the solution from yellow to green ([Fe(CN)_6_]^3−^) to colourless ([Fe(CN)_6_]^4−^). The ability to continuously utilise [Fe(CN)_6_]^3−^ as an electron acceptor was further tested in the BESs, where electrochemical oxidation of the [Fe(CN)_6_]^4−^ at the anode allowed constant regeneration of the [Fe(CN)_6_]^3−^ for microbial metabolism. In the absence of a mediator, *P. putida* F1 did not transfer electrons to the anode, since no catalytic current was detected when no mediator was added (Additional file 1: Fig. S4).

The combination of the BES and [Fe(CN)_6_]^3−^ as electron acceptor proved to be a feasible solution to the problem of electron transport. *P. putida* F1 was able to perform detectable anoxic metabolic conversions in the BES with an applied potential of +0.697 V vs SHE compared to the control, as indicated by a drop in medium pH (Fig. 1b). The total concentration of [Fe(CN)_6_]^3−^ and [Fe(CN)_6_]^4−^ quantified by a developed optical method (Additional file 1: Fig. S5), was constant during the whole operating batch, confirming this chemical only served as electron mediator. The pH drop from 7.06 to 5.84 pointed towards the production of acids due to the metabolism of glucose [46]. The measured catalytic current (max current 0.066 mA cm^−2^) confirmed electrons were released from *P. putida* F1 to the anode. This electron flux is indicative of the anoxic catabolism of glucose since this was the only electron donor in the system at sufficiently high concentration to induce a transfer of charge of over 850 °C in 218 h. The anode chamber contained 2.55 mmol glucose, and around 10 mmol electrons were released over the course of the experiment. Providing an external electron sink to the cell in the form of an anode and a mediator molecule enabled the wild-type *P. putida* F1 to stay metabolically active for over 300 h under anaerobic conditions. The concentration of planktonic cells decreased over time similar to the controls lacking the anode (Fig. 1a), but a non-homogeneous biofilm formation (unquantified) on the carbon cloth electrode could be observed during the electrochemical experiments (Additional file 1: Fig. S6). This not only explains, on the one hand, the drop in planktonic cells but also indicates that anaerobic growth may have been possible to a limited extent. Metabolic engineering was previously used to adapt *P. putida* to anaerobic conditions by aiming at balancing the energy and redox couples [18], but in that study, only the death rate could be reduced, while metabolic turnover remained low.

### Biochemical production in the presence of different mediators

After confirming that providing an extracellular electron sink to *P. putida* F1 in the form of electrodes and redox mediators results in production of organic acids, additional experiments were performed with the aims (1) to determine the complete product spectrum and (2) to describe the fermentation kinetics in relation to the redox potential of the mediator, through testing of compounds covering a broad range of redox potentials between approximately −0.4 and +0.4 V.

**Fig. 2:**
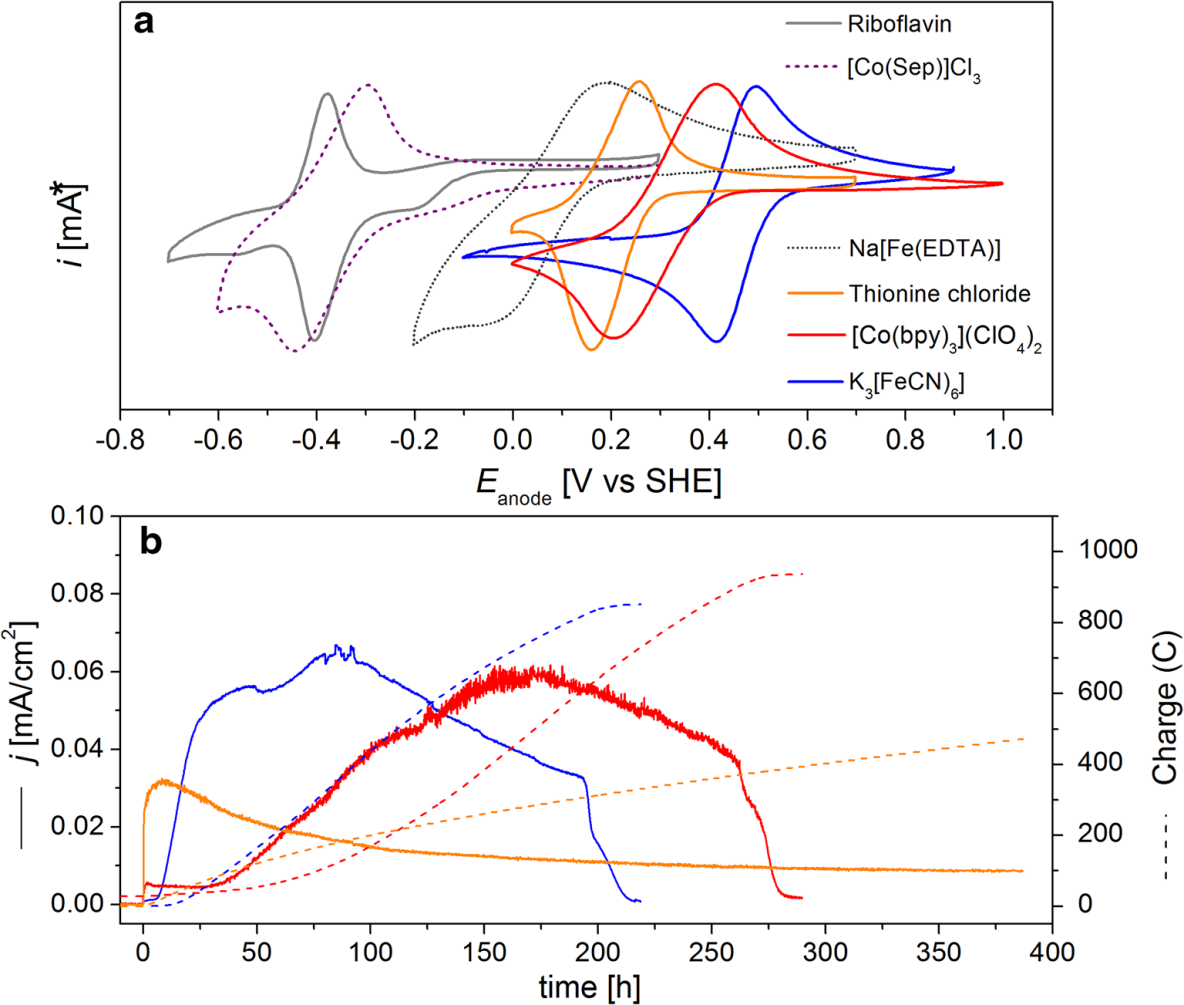
Electrochemical characterisation of the redox mediators used in this study by cyclic voltammetry (*CV*) (**a**); anodic current (*solid line*) and charge (*dash line*) production measured in the presence of mediators that show activity with *P. putida* (**b**). *I [mA]: each cyclic voltammogram is shown in its *optimum scale* to give a clear appearance for all compounds

Cyclic voltammetry was used to determine the average midpoint potential values (*E*_m_) of the mediators used in this study. Characteristic CV traces at the scan rate of 50 mV s^−1^ are reported in Fig. 2a while *E*_m_ values are listed in Table 1. The measurements confirmed the relatively low midpoint potential of riboflavin and [Co(Sep)]Cl_3_, centred at around −0.365 and −0.349 V, respectively, whereas Fe(EDTA), thionine chloride, [Co(bpy)_3_](ClO_4_)_2_ and K_3_[Fe(CN)_6_] displayed more positive *E*_m_, centred at around 0.078, 0.208, 0.310, and 0.416 V, respectively. These values are in good agreement to those reported previously for the same compounds (e.g., see references [47–52]) thus confirming that the electrode material used in this study (carbon cloth) was suitable for the electrochemical conversion of the redox mediators tested.

**Table 1:** Formal redox potential of tested mediator molecules and their interaction with *P. putida* F1

Redox chemical	*E*_m_ = (*E*_pa_ + *E*_pc_)/2 [V] vs SHE	Interact with *P. putida* F1
Riboflavin	−0.365	No
[Co(Sep)]^3+/2+^	−0.349	No
[Fe(EDTA)]^−/2−^	0.078	No
Thionine chloride	0.208	Yes
[Co(bpy)_3_]^3+/2+^	0.31	Yes
[Fe(CN)_6_]^3−/4−^	0.416	Yes

**Fig. 3:**
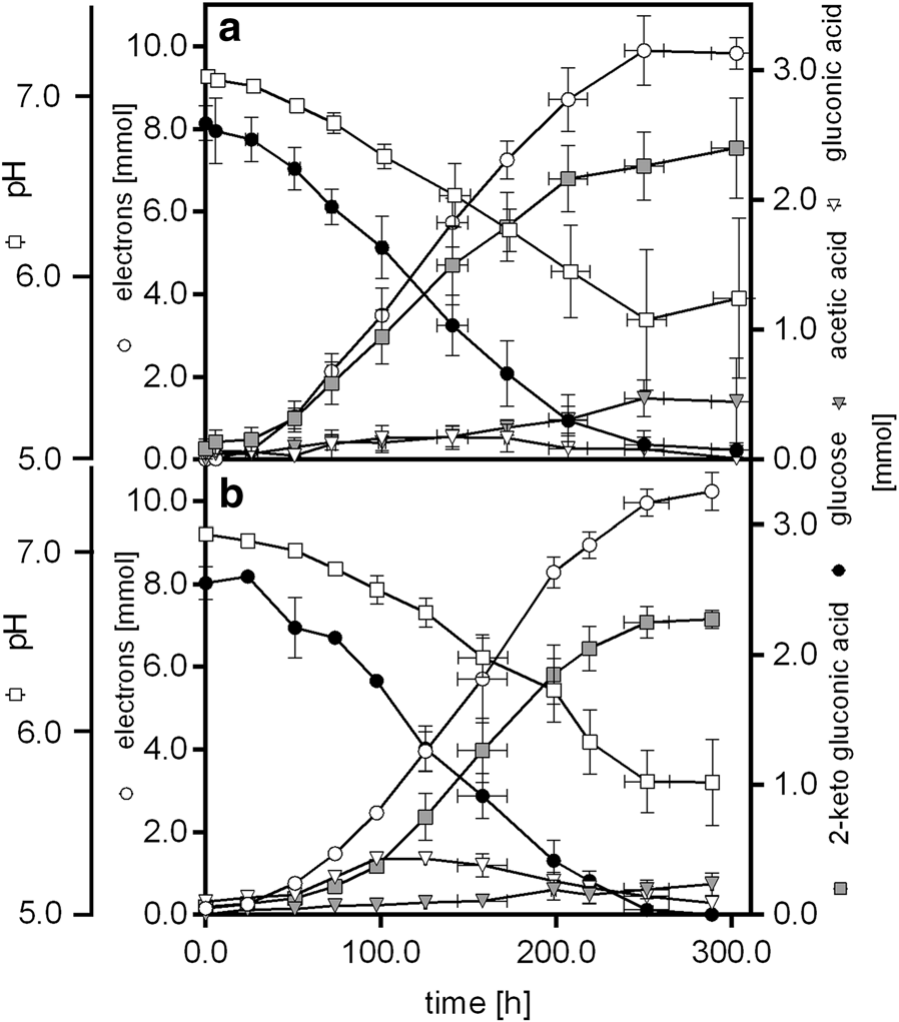
Total metabolite levels and pH in the anode compartment of the BES reactors with K_3_[Fe(CN)_6_] (**a**) and [Co(bpy)_3_](ClO_4_)_2_ (**b**) as mediators, respectively. The cumulative amount of electron produced during the conversions is also indicated. Data have been averaged from 10 (**a**) and four (**b**) biological replicates with a total of 79 and 36 samples, respectively. Means and standard deviations are given [average sample size in each point *n* = 7 (**a**); *n* = 3 (**b**)]

Neutral red [53, 54] and riboflavin [55, 56] have been previously used successfully in combination to organisms such as *E. coli* and *Shewanella oneidensis* to shuttle electrons extracellularly by coupling with the respiratory chain using NADH as the electron donor. However, when we added these mediators to the cultures of *P. putida* F1, no significant current production was observed (Additional file 1: Fig. S4), as was for [Co(Sep)]Cl_3_ and Na[Fe(EDTA)]. On the contrary, mediators with redox potentials above 0.207 V, that is thionine chloride, [Co(bpy)_3_](ClO_4_)_2_ and K_3_[Fe(CN)_6_], demonstrated the ability to accept electrons from *P. putida* F1 cells (Fig. 2b). In fact, current output (Fig. 2b), glucose consumption and a drop in pH (Fig. 3) were observed when these mediators were present in the culturing solutions.

The catalytic current increased over time (Fig. 2b), despite the planktonic biomass concentration was stable after the drop during the initial 24 h. We hypothesise that the observed slow formation of a biofilm on the anode is the likely explanation for this increase along with the possibility of a change in gene expression of relevant membrane proteins involved in the mediated electron transport. Depending on available energy, both could be very slow processes.

HPLC analysis showed also the consumption of glucose by *P. putida* in the presence of each of the three mediators. However, in the case of thionine chloride, conversion rates were very low and no full substrate conversion could be reached within 400 h (Fig. 2b). Therefore, only production with [Co(bpy)_3_](ClO_4_)_2_ and K_3_[Fe(CN)_6_] was analysed in detail. For easier comparison with the produced electrons, concentrations were converted to absolute moles using the respective reactor volumes at each time point. Glucose was converted into three detectable products: 2KGA, gluconic acid and acetic acid with the former being the dominant product (Fig. 3). Gluconic acid was accumulated in the first 100 h of the cultivations and then consumed. This phenotype was much more pronounced in the case of [Co(bpy)_3_](ClO_4_)_2_ where not only a higher amount of gluconic acid was observed in transition, but also a net production remained at the end of the experiment. In the case of the K_3_[Fe(CN)_6_]-mediated electron transport, all gluconic acid was consumed in the second half of the fermentation, and acetate production was also higher in this process (Fig. 3).

The carbon balances closed in both studies (Table 2) under the assumption that per mol of acetic acid, one mol of CO_2_ would be released in metabolism (see M&M section). For both mediators, it was found that around 90 % of the glucose was converted to 2KGA, while twice as much acetic acid accumulated with K_3_[Fe(CN)_6_] compared to [Co(bpy)_3_](ClO_4_)_2_ (Table 2). The yields of electrons produced from glucose were comparable with both mediators, but the current profiles (Fig. 2b) indicated that the conversion rates were higher in the case of K_3_[Fe(CN)_6_]. While it is not possible to quantify the exact amounts of cells growing on the electrodes, the carbon balance indicated that growth would be minimal, which is also in agreement with our visual observations. Using the average planktonic biomass concentration over the course of the processes it was possible to calculate specific rates (Table 2). This shows that in fact the bioconversion in the presence of K_3_[Fe(CN)_6_] was much faster and was characterised by a glucose consumption rate that was 36 % higher compared to [Co(bpy)_3_](ClO_4_)_2_ and the same holds true for the rate of electron production. This rate was positively correlated to the redox potential of the mediators used; the more positive the potential, the faster the production (Table 2, Fig. 2b). Qualitatively this is expected according to Marcus’ theory of electron transfer kinetics [57–59]. These results indicate that the anoxic metabolism was driven by the capability of the redox mediators to scavenge electrons from the intracellular metabolism of *P. putida* F1, since increasing conversions rates could be observed with mediators with higher potentials. Interestingly, the current density recorded immediately after the inoculation of microbes showed a different trend from the production rate of products: thionine chloride and [Co(bpy)_3_](ClO_4_)_2_ could trigger electron transfer to the anode more rapidly than K_3_[Fe(CN)_6_] in spite of having a lower redox potential than the latter (0.208 V and 0.31 V, respectively, vs 0.416 V of ferricyanide) (Fig. 2b). A similar change was also observed for coulombic efficiency, as the reactor with [Co(bpy)_3_](ClO_4_)_2_ gave a higher value than when K_3_[Fe(CN)_6_] was used (Table 2), showing that in the faster process using K_3_[Fe(CN)_6_] more electrons are lost. Despite these losses, however, it emerges that a BES can be used to produce oxidised products under oxygen-free conditions at high yield, high purity and with minimal production of biomass, and (as it is the case here) without genetic modifications.

**Table 2:** Key process parameters of anaerobic glucose conversion of *P. putida* F1 in the anode compartment of a BES using [Co(bpy)_3_]^3+/2+^ or [Fe(CN)_6_]^3−/4−^ as electron acceptors with the anode potential poised at +0.697 V vs SHE

	[Co(bpy)_3_]^3+/2+^	[Fe(CN)_6_]^3−/4−^
Carbon balance (%)	99.6	97.6
Coulombic efficiency (%)	98.5	93.3
Yields [mol_product_/mol_glucose_]		
*Y*_2KGA_	0.90 ± 0.03	0.90 ± 0.02
*Y*_acetic acid_	0.07 ± 0.01	0.14 ± 0.01
*Y*_gluconic acid_	0.31 ± 0.06	0.09 ± 0.03
	0.25 ± 0.03	0.09 ± 0.04
*Y*_electrons_	3.94 ± 0.11	3.88 ± 0.07
Rates [mmol/(gCDW h)]		
*r*_glucose_	−0.81 ± 0.14	−1.10 ± 0.21
*r*_acetic acid_	0.06 ± 0.01	0.16 ± 0.03
*r*_2KGA_	0.73 ± 0.13	0.99 ± 0.19
*r*_gluconic acid_	0.25 ± 0.06	0.10 ± 0.04
	−0.20 ± 0.04	−0.10 ± 0.05
*r*_electrons_	3.18 ± 0.55	4.28 ± 0.82

Data are fitted with linear regression using datasets from ten ([Fe(CN)_6_]^3−/4−^) and four ([Co(bpy)_3_]^3+/2+^) biological replicates with a total of 79 and 36 samples, respectively (compare Additional file 1: Fig. S3). Carbon balance is calculated from the fitted rates considering carbon content of molecules and assuming equimolar CO_2_ production when making acetate from glucose. Gluconic acid is a product in the first 100 h and serves as a substrate thereafter; hence, 2 yields and rates are given

### Intracellular electron and energy carriers during BES-driven glucose oxidation

The previous sections showed that only redox molecules whose electrochemical potentials were above 0.207 V could successfully shuttle electrons from microbes to the anode (Fig. 2a; Table 1). This potential is positive enough to oxidise a wide range of cellular redox carriers and proteins involved in the electron transport chain of *P. aeruginosa* [60], which has high similarity to the one of *P. putida*. This should enable further oxidation of carbonaceous matter, instead, our results shows that 90 % of the carbon provided accumulated as 2KGA in the BES, indicating that there is still a metabolic constraint in the cells that prevents the full utilisation of the carbon source under anaerobic conditions. In fact, no obvious growth could be observed. This is somewhat surprising, since the accumulation of acetic acid also indicated that some carbon was processed through glycolysis. This imbalance could potentially be explained by a limitation of ATP generation or imbalances of the intracellular redox couples NAD(P)^+^/NADPH.

**Fig. 4:**
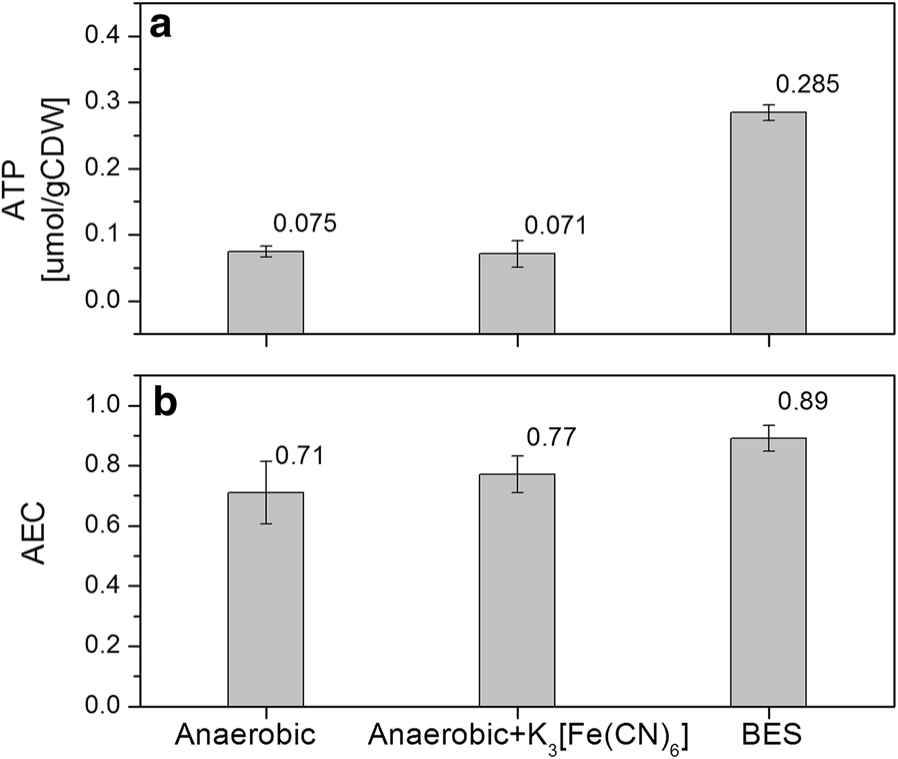
**a** Specific intracellular ATP concentration in *P. putida* F1 under anaerobic conditions in the absence or presence of K_3_Fe(CN)_6_ and electrodes. **b** Adenylate energy charge under the same conditions (AEC = (ATP + 0.5×ADP)/(ATP + ADP + AMP))

To shed light on these possible limitations, analyses of intracellular concentrations of NADH, NAD^+^, NADPH, NADP^+^, ATP, AMP and ADP were performed for the experiments using the best performing mediator K_3_[Fe(CN)_6_] (Fig. 3a). Concentrations of intracellular ATP were compared to cells incubated in identical medium with and without K_3_[Fe(CN)_6_] in an anaerobic chamber. The intracellular ATP concentration in the BES was much higher than in the anaerobic conditions without an anode provided as electron acceptor (Fig. 4a). While we could not find data on anaerobic cultures of *P. putida*, the determined intracellular ATP concentrations are below 10 % of the ranges published for aerobically growing *P. putida* strains [17, 61], but well in agreement with the observed concentrations during carbon starvation [62] (note that concentrations were converted assuming 1.19 × 10^^^12 cfu/g_CDW_ [63]). When comparing the adenylate energy charge (AEC), which uses the ratio of ATP, ADP and AMP to estimate the relative amount of energy rich phosphate bonds, it could be observed that providing the anode and the mediator in the BES helped the cells to restore the AEC to 0.9 (Fig. 4b). The AEC should be maintained normally over 0.8 for growing microbes [44, 64] and for *P. putida* under aerobic conditions, and exponential growth values between 0.75 and 0.95 have been described [61, 65]. These findings indicate that the BES could potentially provide enough energy for growth, but carbon turnover seems to be limiting.

**Fig. 5:**
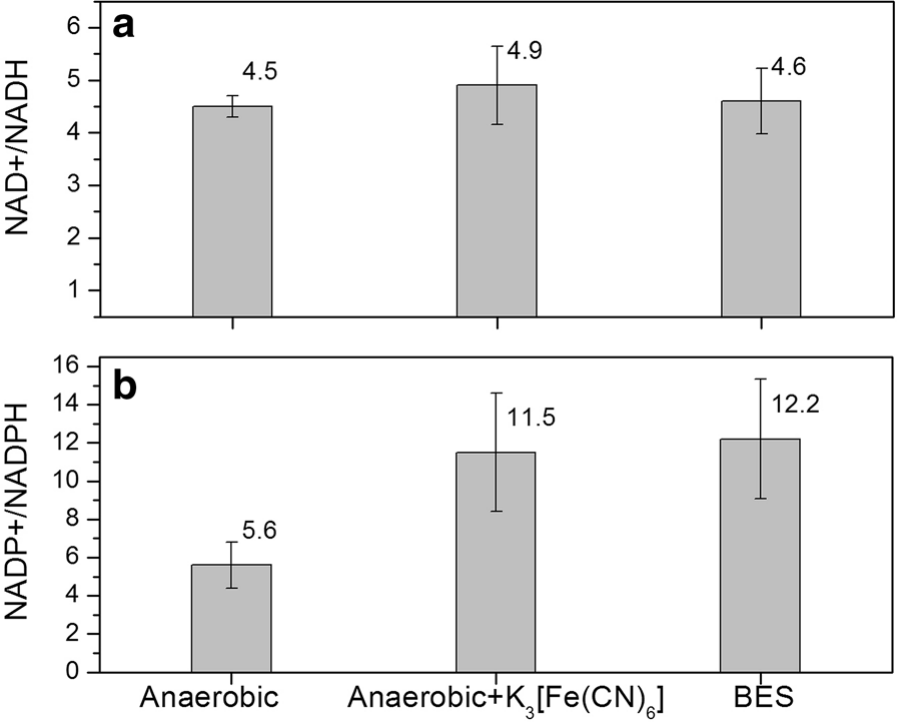
Determination of intracelluar pyridine nucleotide cofactors in *P. putida* under anaerobic conditions in the absence or presence of K_3_[Fe(CN)_6_] and electrodes. Analytical samples were taken using the same procedures as those for ATP determination

When analysing the intracellular concentrations of redox cofactors it was observed that the ratios of NAD^+^/NADH and NADP^+^/NADPH were shifted towards the oxidised species under all conditions (Fig. 5). The ratio of NAD^+^/NADH remained, however, similar for the three tested conditions. The observed ratios for NAD^+^/NADH are highly similar to that observed for aerobic *P. putida* KT2440 [65], which is somewhat surprising since one would expect that in the absence of an electron acceptor (anaerobic control Fig. 5a) the NADH pool would be more reduced (It is important to note that the inverse ratio is given in the cited reference). These data show, however, that adding (electro)chemical oxidants did not alter overall NAD^+^/NADH balance. This situation was quite different for the couple NADP^+^/NADPH. In fact, the presence of oxidised mediators increased the ratio significantly (Fig. 5b), showing that the NADPH pool became more oxidised. The anaerobic control exhibited a NADP^+^/NADPH ratio which is already more oxidised than in the case of aerobically growing *P. putida* KT2440 [65]. This could point to a limitation in the activity of the PPP for NADPH regeneration, and the imbalance of NADP^+^/NADPH is aggravated by the presence of mediator and by the use of a BES. This imbalance may hamper the uptake and processing of carbon on the level of 2KGA and could explain the ATP concentrations that point towards carbon starvation.

### Flux balance analysis

**Fig. 6:**
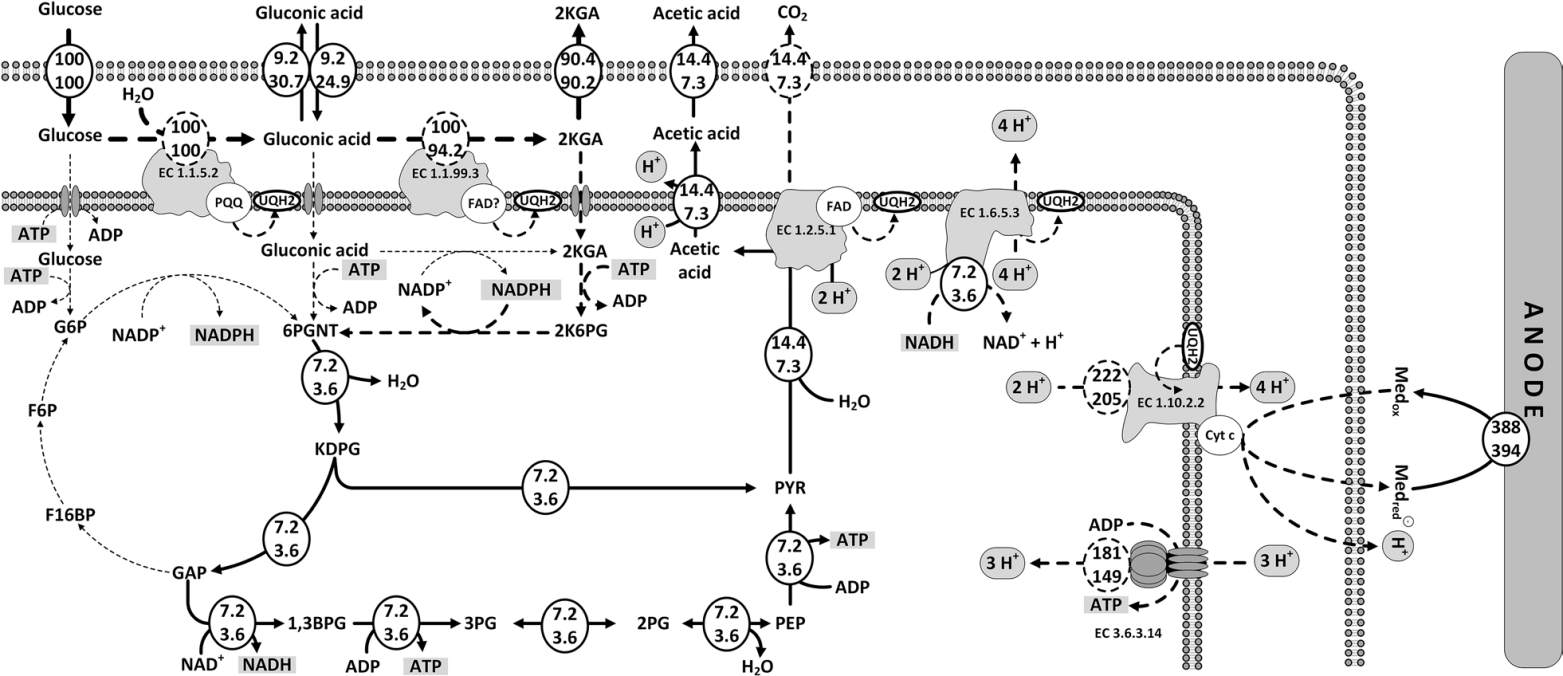
Estimated flux distribution in *P. putida* F1 during glucose oxidation in the anode compartment of the BES reactors with K_3_[Fe(CN)_6_] (numbers on *top*) and [Co(bpy)_3_](ClO_4_)_2_ (numbers on *bottom*) as mediators, respectively. *Solid lines* represent measured fluxes and fluxes derived from mass balancing. *Dashed lines* highlight assumed fluxes, not directly deducible from mass balancing. *PQQ* pyrroloquinolinequinone, *FAD* flavin adenine dinucleotide, *UQH2* reduced ubiquinones, *Cyt C* cytochromes C, *ADP* adenosine diphosphate, *ATP* adenosine triphosphate, *NADP* ^*+*^ */NADPH* nicotinamide adenine dinucleotide phosphate (oxidised / reduced), *NAD* ^*+*^ */NADH* nicotinamide adenine dinucleotide (oxidised / reduced), *2KGA* 2-keto-gluconic acid, *2K6PG* 2-keto-gluconic acid-6-phosphate, *6PGNT* 6-phosphogluconic acid, *KDPG* 2-keto-3-deoxy-phosphogluconic acid, *GAP* glyceraldehyde-3-phosphate, *F16BP* fructose-1,6-bisphosphate, *F6P* fructose-6-phosphate, *G6P* glucose-6-phosphate, *1,3BPG* 1,3-biphosphoglyceric acid, *3PG* 3-phosphoglyceric acid, *2PG* 2-phosphoglyceric acid, *PEP* phosphoenolpyruvic acid, *PYR* pyruvic acid, *Med*_*ox*_ oxidised mediator, *Med*_*red*_ reduced mediator

Using existing knowledge of the underlying pathway stoichiometry and the measured rates from the previous sections, it is possible to conduct a flux balance analysis using a simplified model (zero growth, conversion of glucose to gluconic acid, 2KGA, acetic acid and CO_2_ and electrons) (Fig. 6). The estimation of fluxes around the uptake of sugar or sugar acids, is complicated by the fact in *P. putida* transporters for the uptake of glucose, gluconic acid and 2KGA are present. We assume that the 2KGA present in the periplasm was the main C_6_ molecule imported into the cytoplasm and base this assumption on the observed shift in the NADPH ratio. This unbalanced consumption of NADPH could have resulted in the high ratio of NADP +/NADPH determined in BES condition (Fig. 5b). This could also be the reason for the changed ratio in the case of anaerobic cultivation adding K_3_[Fe(CN)_6_], because the small pool size of NADPH can experience a redox shift due to the mediator accepting electrons without a measurable difference in extracellular substrate concentrations.

The observed increase in AEC (Fig. 4) raises the question if the energy is generated through a proton gradient-driven ATP synthase and/or substrate level phosphorylation. In the case of both mediators, acetic acid could be observed as a minor by-product derived from glycolytic pathways, through which ATP could be generated on the level of phosphoglycerate kinase (Fig. 6). The reported non-growth associated maintenance (NGAM) demand for *P. putida* KT2440 has been reported to be between 0.92 mmol_ATP_/(g_CDW_ h) [16] and 3.96 mmol_ATP_/(g_CDW_ h) [66]. Since the estimation of NGAM requires growth [67] it is currently not feasible to determine it in the BES. Independent of the assumed C_6_ uptake system, one mol ATP will be required for kinase reactions, while two mol ATP will be generated in glycolysis per mol C_6_ substrate taken up (Fig. 6). Based on the rates for acetate production (Table 1) and due to the fact that *P. putida* does not possess an acetate kinase [18] this equates to 0.06 and 0.16 mmol_ATP_/(g_CDW_ h) for [Co(bpy)_3_](ClO_4_)_2_ and K_3_[Fe(CN)_6_], respectively, or 1.5–6.5 % and 4–17 % of NGAM, depending which literature value is assumed. Even if the lower value for NGAM is still an overestimation, it seems that an additional energy production must be present, to explain the ongoing metabolic activity of the cells. Since carbon balances were closed and no other products detectable, this indicates that the cells must be able to generate energy through a process coupled to the electron transport process. Under the common assumption that three protons move through the ATP synthase to generate one ATP [68] and further respecting charge balance, which means that each of the electrons donated to the anode (measured as current) would have to take a cation (proton) out of the periplasmic space for charge balance, it is possible to balance the available periplasmic protons for ATP synthase (EC 3.6.3.14). For simplicity and due to the lack of knowledge, we assume that all electron transport is mediated via the quinone pool and that the mediator interacts with the terminal oxidase (EC 1.10.2.2) containing cytochrome *c*. This is thermodynamically feasible, based on the observed redox potentials (Table 1) [60]. The fact that the NAD +/NADH ratio remains on the oxidated side (Fig. 5) also implies that the NADH generated through acetic acid synthesis (Fig. 6) could be re-oxidised through the NADH dehydrogenase complex (EC 1.6.5.3) (Fig. 6) and hence the quinone pool must be re-oxidised through the interaction with the mediator.

Using the flux analysis to estimate the rate of ATP synthesis through ATP synthase that could be derived through the available proton gradient shows that 1.2 and 1.3 mmol_ATP_/(g_CDW_ h) for [Co(bpy)_3_](ClO_4_)_2_ and K_3_[Fe(CN)_6_], respectively, could have been generated. This would bring the total energy available within the range of NGAM, making this scenario much more likely than energy production through acetic acid formation alone. While this is only an estimation, it supports the idea that anodic oxidation of carbohydrates in a BES could also lead to ATP generation in the cells, which in our opinion is a prerequisite for maintaining active bio-catalysts over long periods of time and crucial for the viability of BES applications for bio-production.

## Conclusions

By providing an electrode and redox chemicals as extracellular electron sinks, wild-type *P. putida* F1 was able to perform anoxic metabolism, without the need for metabolic engineering and without the formation of biomass as a substrate draining by-product. The redox power from electrode and redox chemicals drove the carbon flux from glucose to 2-Keto-gluconate with a high yield of over 90 %. A survey of different redox chemicals showed that a redox potential of above 0.207 V was crucial and that reaction rates increased with increasing redox potential. Energy was generated in metabolism, but the cells remained largely unable to fully oxidise the substrates to CO_2_ despite the intracellular redox co-factors being mainly oxidised. However, the study provides a proof of principle that a BES-driven bioconversion of glucose can achieve high yields, high purity and also deliver necessary energy for cell maintenance, and enables a strict aerobe to catalyse production under oxygen-free conditions for over a week. This opens the route to bi-phasic bio-processes, where the catalyst is grown under aerobic conditions and then used for anaerobic catalysis over long periods of time, without observable growth and hence drain of substrate. Combining this with metabolic engineering strategies could prove to be a powerful new way to produce bio-chemicals from renewable materials.

## Additional file

10.1186/s13068-016-0452-y Picture and the schematic drawing of BES reactor used. **Fig. S2.** Inhibition of Trichloroacetic acid (TCA) on the bioluminescent assay for ATP determination. **Fig. S3.** Regression analysis for the determination of product/glucose yield coefficients. **Fig. S4.** Current–time curve for BES reactors with/without mediators. **Fig. S5.** Determination and quantification of K_3_[Fe(CN)_6_] and K_4_[Fe(CN)_6_] by UV–vis spectroscopy. **Fig. S6.** Biofilm observed on the anode of BES reactor. **Fig. S7.** Abiotic control with mediator and full medium under BES conditions.

## Abbreviations

ADP: adenosine diphosphate
AEC: adenylate energy charge
AMP: adenosine monophosphate
ATP: adenosine triphosphate
BES: bioelectrochemical system
CB: carbon balance
CDW: cell dry weight
CE: coulombic efficiency
CTAB: cetrimonium bromide
CV: cyclic voltammetry
Cyt C: cytochromes C
DM9: defined mineral medium
EDTA: ethylenediaminetetraacetic acid
FAD/FADH_2_: flavin adenine dinucleotide
F16BP: fructose-1,6-biphosphate
F6P: fructose-6-phosphate
GAP: glyceraldehyde-3-phosphate
G6P: glucose-6-phosphate
HPLC: high performance liquid chromatography
KDPG: 2-keto-3-deoxy-phosphogluconic acid
Med_ox_: oxidised mediator
Med_red_: reduced mediator
NAD^+^/NADH: nicotinamide adenine dinucleotide (oxidised/reduced)
NADP^+^/NADPH: nicotinamide adenine dinucleotide phosphate (oxidised/reduced)
NGAM: non-growth-associated maintenance
PEP: phosphoenolpyruvic acid
PQQ/PQQH_2_: pyrroloquinoline quinone
PYR: pyruvic acid
SHE: standard hydrogen electrode
TCA: trichloroacetic acid
UQH2: reduced ubiquinones
1,3BPG: 1,3-biphosphoglyceric acid
2KGA: 2-keto-gluconic acid
2K6PG: 2-keto-gluconic acid-6-phosphate
2PG: 2-phosphoglyceric acid
3PG: 3-phosphoglyceric acid
6PGNT: 6-phosphogluconic acid
