# Erratum to: Anoxic metabolism and biochemical production in *Pseudomonas putida* F1 driven by a bioelectrochemical system

**DOI:** 10.1186/s13068-017-0843-8

**Published:** 2017-06-19

**Authors:** Bin Lai, Shiqin Yu, Paul V. Bernhardt, Korneel Rabaey, Bernardino Virdis, Jens O. Krömer

**Affiliations:** 1 Centre for Microbial Electrochemical Systems (CEMES), The University of Queensland, Office 618, Gehrmann Building (60), St. Lucia, Brisbane, QLD 4072 Australia; 2 Advanced Water Management Centre (AWMC), The University of Queensland, Brisbane, Australia; 3 School of Chemistry and Molecular Biosciences, The University of Queensland, Brisbane, Australia; 4 Laboratory of Microbial Ecology and Technology (LabMET), Ghent University, Ghent, Belgium

## Erratum to: Biotechnol Biofuels (2016) 9:39 DOI 10.1186/s13068-016-0452-y

After the publication of the article [1], it was brought to our attention that some of the data in Table 2 were incorrect. Please find a correct and updated version of Table 2 in the erratum. Following this Fig. 1 has also been updated; the correct version of Fig. 1 is given in this erratum.

**Table 2:** Key process parameters of anaerobic glucose conversion of *P. putida* F1 in the anode compartment of a BES using [Co(bipy)_3_]^3+/2+^ or [Fe(CN)_6_]^3−/4−^ as electron acceptors while poising the anode at +0.697 V vs SHE

	[Co(bipy)_3_]^3+/2+^	[Fe(CN)_6_]^3−/4−^
Carbon balance (%)	99.6	97.6
Coulombic efficiency (%)	98.5	93.3
Yields (mol_product_/mol_glucose_)		
*Y*_2KGA_	0.90 ± 0.03	0.90 ± 0.02
*Y*_acetic acid_	0.073 ± 0.008	0.144 ± 0.012
*Y*_gluconic acid_	0.31 ± 0.06	0.09 ± 0.03
	0.25 ± 0.03	0.09 ± 0.04
*Y*_electrons_	3.94 ± 0.11	3.88 ± 0.07
Rates (mmol/(gCDW h))		
*r*_glucose_	−0.26 ± 0.04	−0.35 ± 0.07
*r*_acetic acid_	0.019 ± 0.003	0.051 ± 0.010
*r*_2KGA_	0.23 ± 0.04	0.32 ± 0.06
*r*_gluconic acid_	0.08 ± 0.02	0.03 ± 0.01
	−0.06 ± 0.01	−0.03 ± 0.02
*r*_electrons_	1.02 ± 0.18	1.37 ± 0.26

Data are fitted with linear regression using datasets from ten ([Fe(CN)_6_]^3−/4−^) and four ([Co(bipy)_3_]^3+/2+^) biological replicates with a total of 79 and 36 samples, respectively (compare Additional file 1: Fig. S3). Carbon balance is calculated from the fitted rates considering carbon content of molecules and assuming equimolar CO_2_ production when making acetate from glucose. Gluconic acid is a product in the first 100 h and serves as a substrate thereafter, hence 2 yields and rates are given

**Fig. 1:**
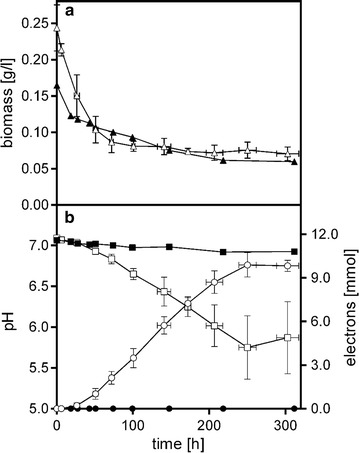
Change of biomass (*triangles*, **a**), pH (*squares*, **b**) and electron production (*circles*, **b**) in the anode compartment of a BES reactor of *P. putida* F1 with K_3_[Fe(CN)_6_] as electron acceptor in control (*black symbols*) and closed circuit with the anode potential poised at +0.697 V (*white symbols*). Data have been averaged from ten (closed circuit) and three (control) biological replicates with a total of 79 and 30 samples, respectively. Means and standard deviations (*X* and* Y* error bars) are given [average sample size *n* = 7 (closed circuit); exact sample size* n* = 3 (control)]

Also, during the calculation of specific glucose uptake rate, the authors mistakenly used the unit mmol/L as mmol, and therefore it caused some errors in the calculations of production rate (Table 2) and ATP regeneration rate (Section “Flux balance analysis”—[1]) which need to be corrected. The corrected ATP regeneration rates are 0.02 and 0.38 mmol_ATP_/(gCDW h) for [Co(bpy)_3_](ClO_4_)_2_ from glucose oxidation and membrane-bound ATP synthase respectively, while those numbers for K_3_[Fe(CN)_6_] are 0.05 and 0.64 mmol_ATP_/(gCDW h), respectively.
