# Supplementary material for: Anoxic metabolism and biochemical production in Pseudomonas putida F1 driven by a bioelectrochemical system
Source: Biotechnol Biofuels. 2016 Feb 18;9:39. doi: 10.1186/s13068-016-0452-y (PMC4758010; doi:10.1186/s13068-016-0452-y)
Supplement: Supplementary file 1 — 10.1186/s13068-016-0452-y Picture and the schematic drawing of BES reactor used. Fig. S2. Inhibition of Trichloroacetic acid (TCA) on the bioluminescent assay for ATP determination. Fig. S3. Regression analysis for the determination of product/glucose yield coefficients. Fig. S4. Current–time curve for BES reactors with/without mediators. Fig. S5. Determination and quantification of K3[Fe(CN)6] and K4[Fe(CN)6] by UV–vis spectroscopy. Fig. S6. Biofilm observed on the anode of BES reactor. Fig. S7. Abiotic control with mediator and full medium under BES conditions. [file 13068_2016_452_MOESM1_ESM.pdf]

Anoxic metabolism and biochemical production in *Pseudomonas putida* F1 driven by a bioelectrochemical system

Bin Lai,[1,2] Shiqin Yu,[1,2] Paul V. Bernhardt,[3] Korneel Rabaey,[4] Bernardino Virdis,[1,2] Jens O. Krömer[1,2*]

[1]Centre for Microbial Electrochemical Systems (CEMES), The University of Queensland, Brisbane, Australia

[2]Advanced Water Management Centre (AWMC), The University of Queensland, Brisbane, Australia

[3] School of Chemistry and Molecular Biosciences, The University of Queensland, Brisbane, Australia

[4] Laboratory of Microbial Ecology and Technology (LabMET), Ghent University, Belgium

Supplementary figures:

Fig.S1	Picture and the schematic drawing of BES reactor used.
Fig.S2	Inhibition of Trichloroacetic acid (TCA) on the bioluminescent assay for ATP determination.
Fig.S3	Regression analysis for the determination of product/glucose yield coefficients.
Fig.S4	Current-time curve for BES reactors with/without mediators.
Fig.S5	Determination and quantification of $K_3[Fe(CN)_6]$ and $K_4[Fe(CN)_6]$ by UV-vis spectroscopy.
Fig.S6	Biofilm observed on the anode of BES reactor.
Fig. S7	Abiotic control with mediator and full medium under BES conditions.

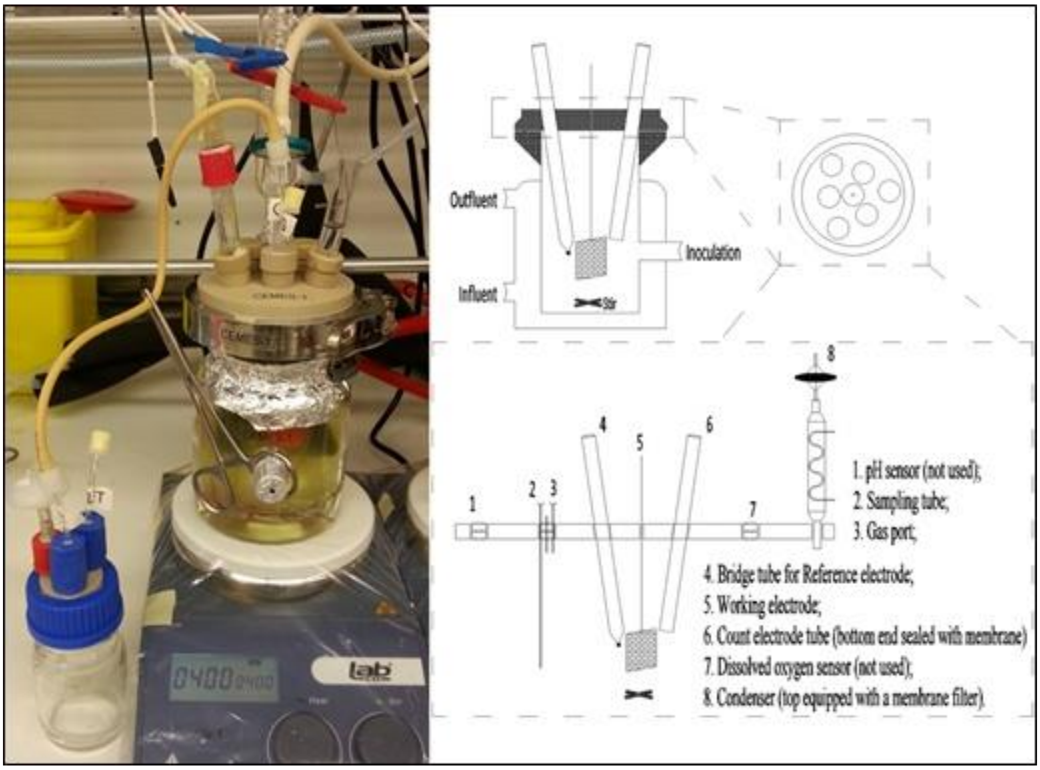

Fig.S1 Picture and the schematic drawing of BES reactor used in this study. The lid was equipped with 7 screw holes designed for the connection of anode, cathode and reference electrode, the control of pH, dissolved oxygen and gas pressure, and do sampling (or continuous flow) respectively.

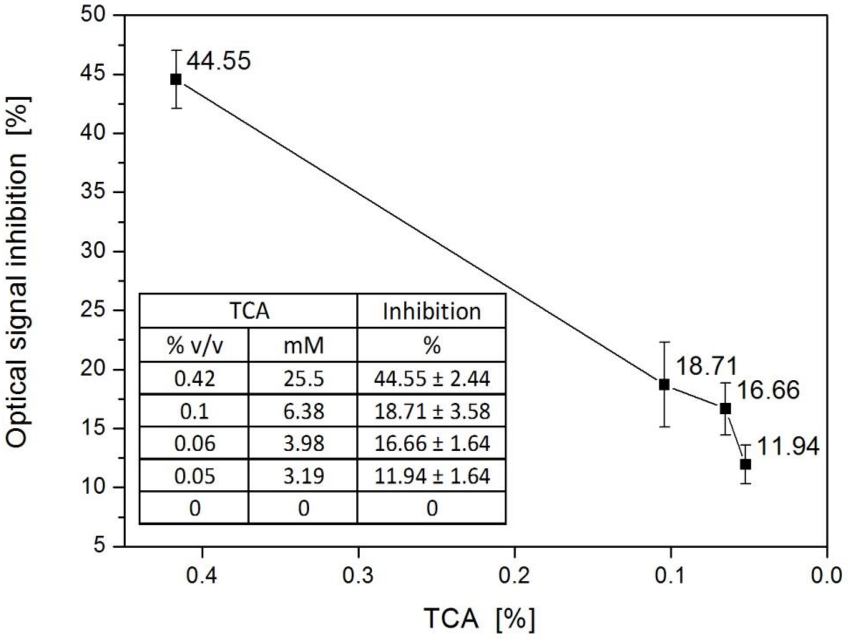

TCA		Inhibition
% v/v	mM	%
0.42	25.5	44.55 ± 2.44
0.1	6.38	18.71 ± 3.58
0.06	3.98	16.66 ± 1.64
0.05	3.19	11.94 ± 1.64
0	0	0

Fig.S2 Inhibition of Trichloroacetic acid (TCA) on the bioluminescent assay for ATP determination.Experiments were tested with ATP standards by adjusting the TCA concentrations in the final assay buffer. Tests were triplicate.

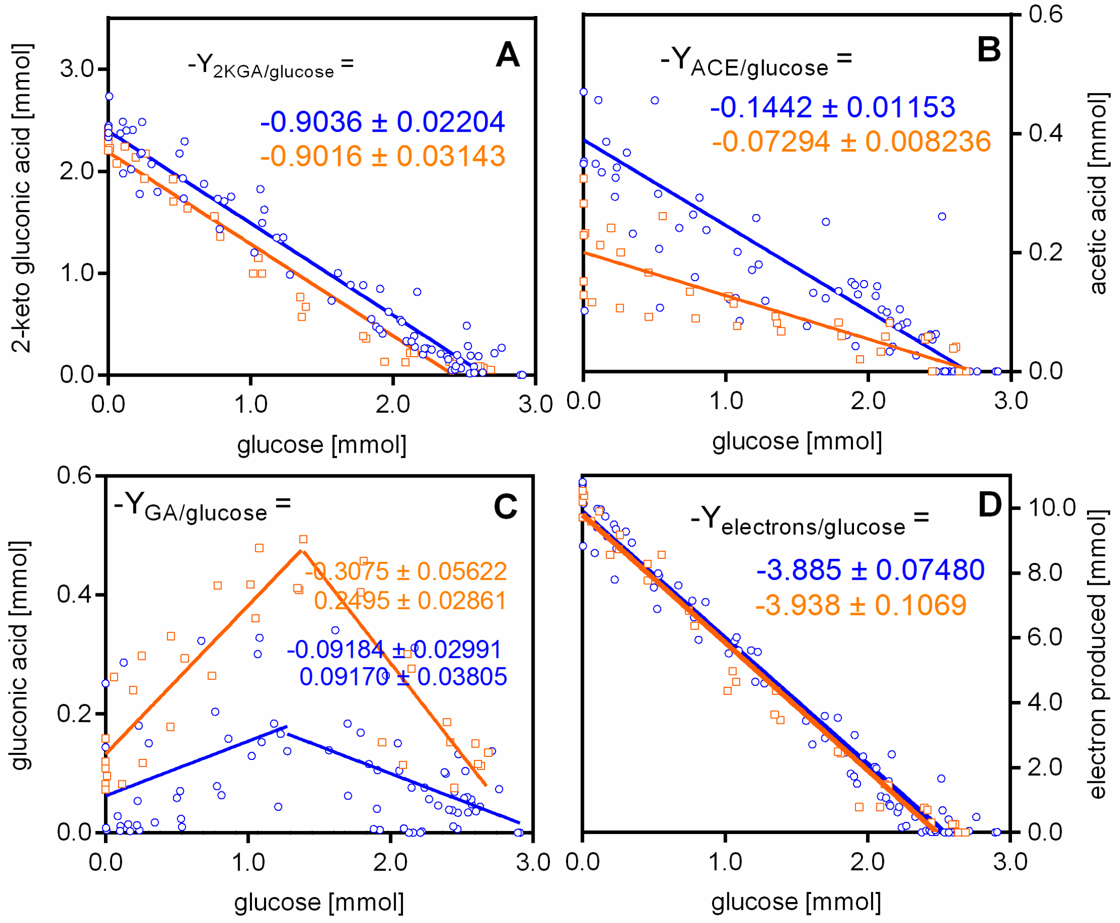

Fig. S3 Regression analysis for the determination of product/glucose yield coefficients. Slope (absolute) of fitted line is the molar coefficient. Analysis performed in Graphpad Prism. A total often ($K_3[Fe(CN)_6]$ BLUE) and four biological replicates ($[Co(bipy)_3](ClO_4)_2$ORANGE) with a total of 79 and 36 samples were used, respectively. Gluconic acid was fitted in two phases, accumulation and consumption. R^2: (A) blue: 0.9562; orange: 0.9603; (B) blue: 0.6699; orange: 0.6976; (C) blue$_{top}$: 0.1765; orange$_{top}$: 0.6971; blue$_{bottom}$: 0.1536; orange$_{bottom}$: 0.7918; (D) blue: 0.9722; orange: 0.9756.

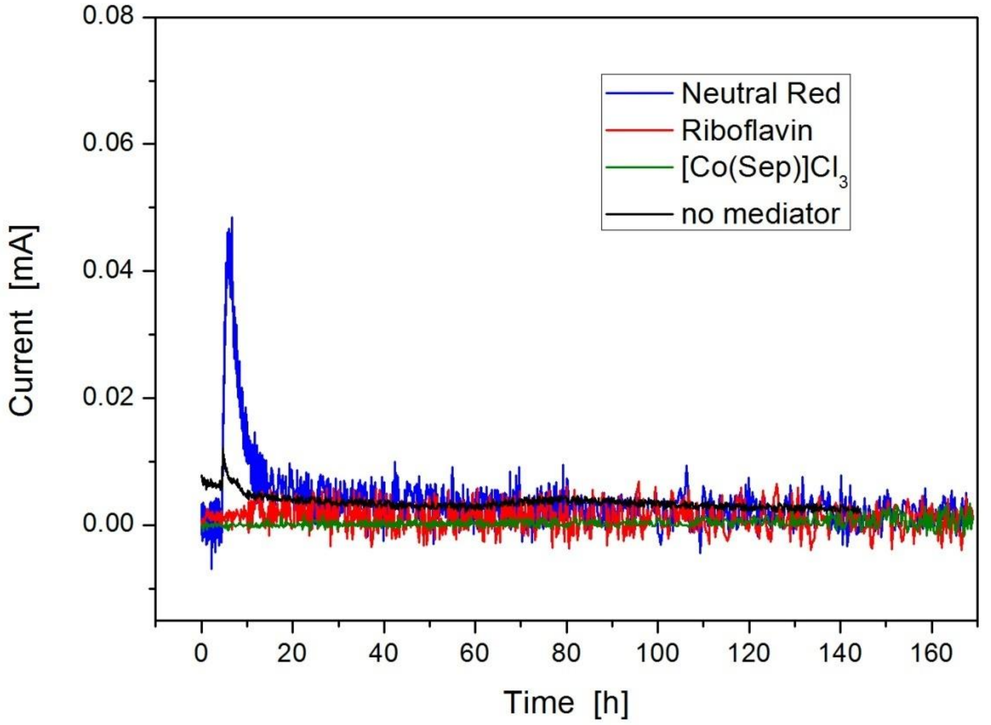

Fig.S4 Current-time curve for BES reactors with/without mediators. All operating parameters are the same as that for reactor with ferricyanide, except adjusting the working electrode potential accordingly. A range of working electrode potential (-0.003-0.797 V vs SHE) was tested for the control (no mediator) and performances were similar under all conditions tested.

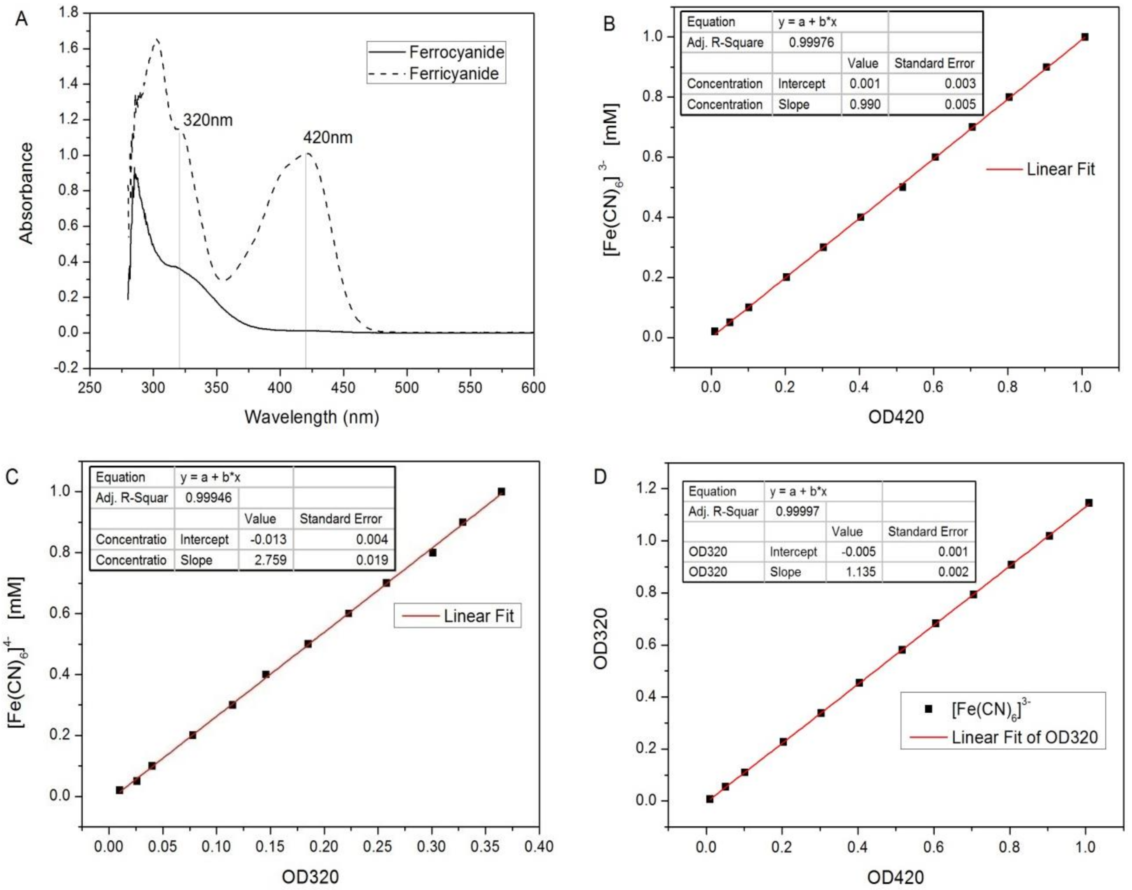

Fig.S5 Determination and quantification of $K_3[Fe(CN)_6]$ and $K_4[Fe(CN)_6]$ by UV-vis spectroscopy. (A) UV-vis spectrum of $K_3[Fe(CN)_6]$ and $K_4[Fe(CN)_6]$; (B) Linear correlations between OD420 and $K_3[Fe(CN)_6]$ concentrations; (C) Linear correlations between OD320 and $K_4[Fe(CN)_6]$ concentrations; (D) Linear correlations between OD320 and OD420 of $K_3[Fe(CN)_6]$.

Based on the UV-vis spectrum (A), OD420 was chosen to quantify the concentration of $K_3[Fe(CN)_6]$. Linear correlation was stated as in Fig. S4(B), and equation is

$$C_{\mathrm{K_3[Fe(CN)_6]}} = 0.990 \times OD420 + 0.001$$

OD320 was chosen to quantify the concentration of $K_4[Fe(CN)_6]$, but since $K_3[Fe(CN)_6]$ also has absorbance at 320nm, this interference has to be subtracted in the calculating equation. As shown Fig.S4(D), the OD320 of $K_3[Fe(CN)_6]$ shows linear relationship with its absorbance at OD420, and therefore, the final equation used to quantify $K_4[Fe(CN)_6]$ could be expressed as:

$$C_{\mathrm{K_4[Fe(CN)_6]}} = 2.759 \times (OD320 - (\,OD420 \times 1.135 - 0.005)) - 0.013$$

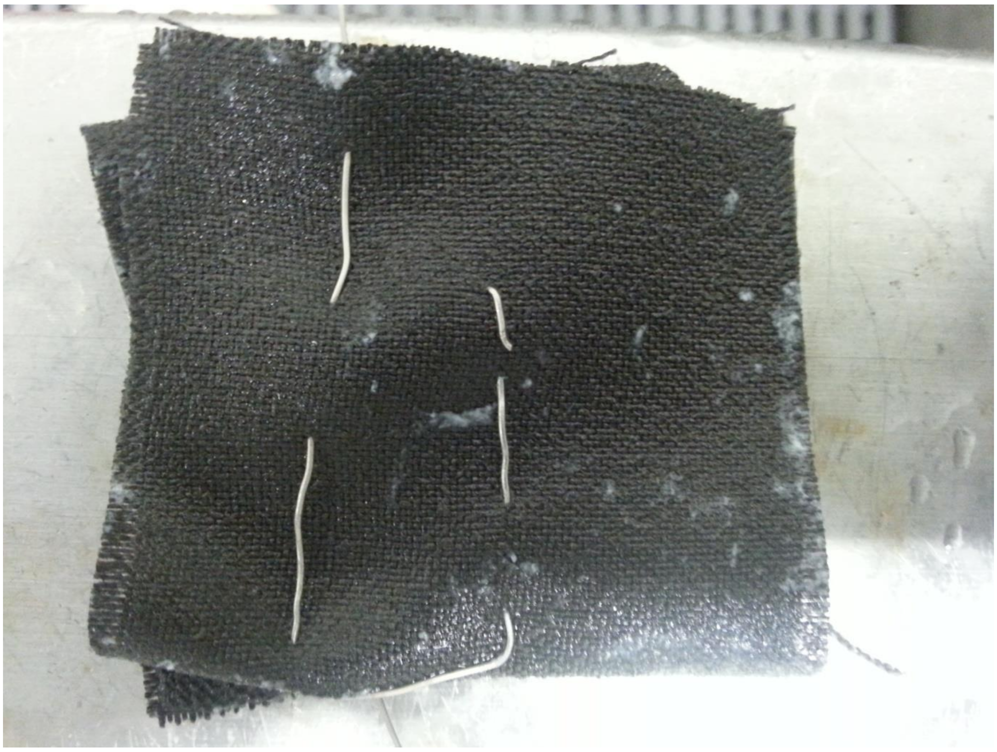

Fig. S6 Biofilm (white spots) observed on the anode of BES reactor

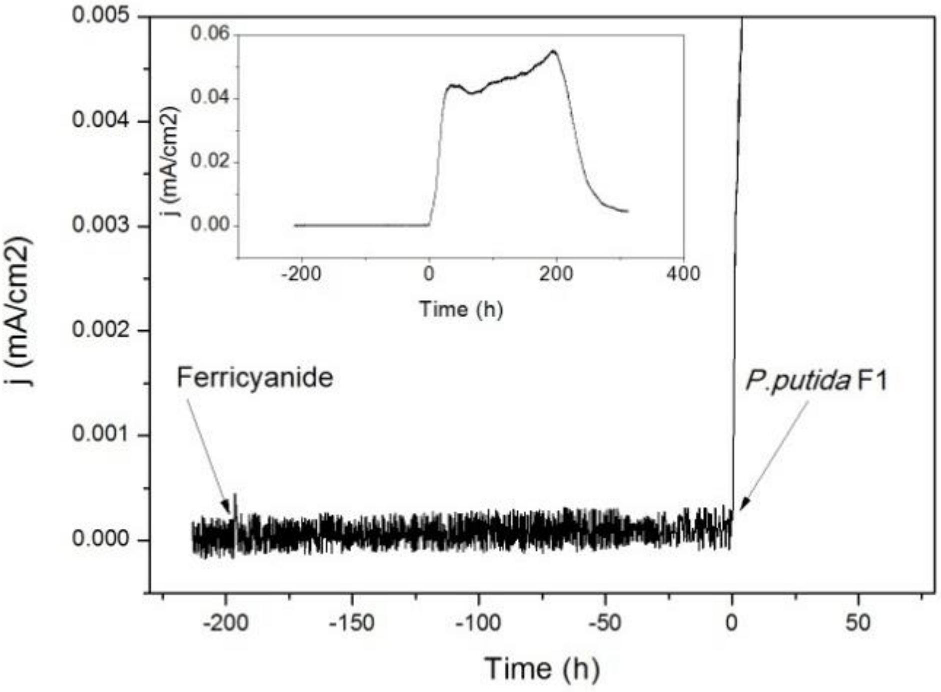

Fig. S7 Absence of current before inoculation with *P. putida* rules out abiotic oxidation of sugar in BES.
